# New Perspective of *Origanum vulgare* L. and *Satureja montana* L. Essential Oils as Bovine Mastitis Treatment Alternatives

**DOI:** 10.3390/antibiotics10121460

**Published:** 2021-11-27

**Authors:** Zorana Kovačević, Nebojša Kladar, Ivana Čabarkapa, Miodrag Radinović, Milan Maletić, Mihajlo Erdeljan, Biljana Božin

**Affiliations:** 1 Department of Veterinary Medicine, Faculty of Agriculture, University of Novi Sad, Trg Dositeja Obradovica 8, 21000 Novi Sad, Serbia; zorana.kovacevic@polj.edu.rs (Z.K.); miodrag.radinovic@polj.uns.ac.rs (M.R.); 2 Center for Medical and Pharmaceutical Investigations and Quality Control, Department of Pharmacy, Faculty of Medicine, University of Novi Sad, Hajduk Veljkova 3, 21000 Novi Sad, Serbia; NEBOJSA.KLADAR@mf.uns.ac.rs (N.K.); BILJANA.BOZIN@mf.uns.ac.rs (B.B.); 3 Institute of Food Technology, University of Novi Sad, Bulevar Cara Lazara 1, 21000 Novi Sad, Serbia; ivana.cabarkapa@fins.uns.ac.rs; 4 Faculty of Veterinary Medicine, University of Belgrade, Bulevar oslobođenja 18, 11000 Belgrade, Serbia; maletic@vet.bg.ac.rs

**Keywords:** antibacterial activity, antibiotics, antioxidant, essential oil, oregano, winter savory, mastitis-causing bacteria

## Abstract

Mastitis represents a heavy burden for the dairy sector worldwide with high economic and animal welfare impact. Antibiotic treatment is an important component of mastitis control programs. However, emergence and transfer of antimicrobial-resistant (AMR) bacteria is becoming a growing concern. Therefore, the development of novel agents is required for prevention and treatment of mastitis. Hence, our aim was to assess the antibacterial properties of two essential oils (EOs) obtained from oregano (*Origanum vulgare* L., Lamiaceae) and mountain savory (*Satureja montana* L., Lamiaceae) against mastitis-associated bacteria in Serbia. The chemical composition and antioxidant potential of these EOs were also evaluated. The present study was conducted on strains derived from aseptic milk samples collected from Holstein-Friesian cows with clinical or subclinical mastitis, during the morning milking. Clinical mastitis was assessed by clinical examination, while subclinical mastitis was confirmed using somatic cell count in the milk samples. The microdilution method was used to determine the antibacterial activity, while antioxidant potential of the EOs was evaluated in several in vitro assays. The values of minimal inhibitory concentrations (MICs) and minimal bactericidal concentrations (MBCs) were used to quantitatively measure the antibacterial activity of each EO. MIC/MBC ranged from 0.78/6.25 and 0.39/0.78 mg/mL for oregano and mountain savory, respectively. A total of 25 compounds were identified in the oregano EO, while 47 were identified in winter savory EO, among which aromatic oxygenated monoterpenes were the most abundant compounds. The tested EOs have shown promising antimicrobial activity and could be considered as one of the treatment approaches in mastitis-affected cows.

## 1. Introduction

As a multifactorial disease, mastitis in dairy cows requires proper herd management to eliminate or minimize its incidence and economic losses due to disease. Additionally, excessive antibiotic treatment of cows with mastitis has resulted in greater resistance of mastitis-associated pathogens [1,2]. This has highlighted the importance of local knowledge of the resistant strains for mastitis treatment solutions. Moreover, the form of mastitis depends on several factors, such as etiology agent, mammary gland response and changes in general health status. From a clinical point of view, two forms of mastitis can occur in dairy cows. A strong immune response to the microorganisms’ activity in the mammary gland leads to the manifestation of clinical mastitis, which is characterized by changes in the udder and the appearance of milk, with a possibility of altered general condition of the animal. There is also subclinical mastitis followed by changes in milk composition and quantity of milk obtained from the affected quarter, but without visible changes in the mammary gland and/or general condition of the animal. Furthermore, several studies claim the frequency of clinical mastitis ranges from 12% to 30% [3], while subclinical mastitis frequency differs between farms, and could be up to 45% in the herd [4]. Subclinical mastitis is proven to be responsible for most mastitis related economic losses, while reduction of milk production accounts for about 70% of total losses [3]. Hence, determination of somatic cell count is a reliable method for diagnosis of subclinical mastitis and is widely recognized as a procedure for control of udder health status changes. It can also be used in milk composition measurement. In milk samples from healthy udders it should not exceed 200,000/mL, while in case of mastitis, somatic cells count will be elevated to over one million [5].

Since public health recognizes milk as an important source of antimicrobial drug-resistant microorganisms which enter the human food chain [6], it is important to find different approaches in mastitis therapy with the aim to avoid excessive use of antibiotics. Lopes, et al. [7] indicated that increasing antimicrobial resistance of pathogens in this disease may affect the efficiency of conventional drugs. With this in mind, essential oils (EOs) are recognized as a possible substitution or addition to conventional antibiotic therapy [8] due to their safety and pharmacological characteristics.

As defined by The International Organization for Standardization (ISO), EOs are “products obtained from a natural raw material of plant origin, by steam distillation, by mechanical processes from the epicarp of citrus fruits, or by dry distillation, after separation of the aqueous phase- if any- by physical processes”. These oils are used in medicine, cosmetics, food and as dietary supplements. Many studies have shown good antimicrobial effects of EOs against the common mastitis pathogens in vitro [9,10]. Additionally, in vivo experiments have demonstrated EOs’ effectiveness in mastitis treatment by improving udder condition, reducing number of somatic cells and elimination of intramammary infection [11].

*Origanum vulgare* (Ov) L., Lamiaceae is a traditional medicinal herb, highly valuable as a spice, and widely used in cosmetics, food and pharmaceutical industry. The chemical composition of oregano EO is, as in many other aromatic plants, highly affected by the biological source used for herbal drug production, phenological stage of development (plants collected before, during, or after flowering period), as well as the range of ecological factors characteristic of habitats [12,13]. The species of another genus of the Lamiaceae family, *Satureja* L., are also widely used as flavoring agents and as traditional medicines due to their content of EOs. Furthermore, the EO from *Satureja montana* (Sm) L., Lamiaceae, commonly called winter or mountain savory, has been recognized for its antimicrobial, antifungal, antioxidant, spasmolytic, antiviral and antidiarrheal activity [14]. It is also widely used as a spice, pepper substitute, and a traditional medicine [15]. Similarly to oregano, *S. montana* is very confusing from taxonomic aspect, with evidently high incidence of polymorphism present, even within a single population, which is reflected in the chemical composition [16]. For both species several chemotypes are described, some of which are characterized by high content of carvacrol [12,16].

So far, published studies on in vitro antimicrobial activity of EOs originating from Serbia mainly examined different foodborne pathogens, such as *S. enteritidis*, *E. coli*, *S. typhimurium*, and *Staphylococcus aureus* [17,18,19], as well fungi [20], but none was focused on bovine mastitis pathogens.

The present study aimed to evaluate in vitro antimicrobial activity of two EOs (*Origanum vulgare* L., Lamiaceae and *Satureja montana* L., Lamiaceae) against strains of mastitis-associated pathogens in Serbia, including important representatives of antibiotic-resistant bacteria, to determine their antibiotic susceptibility patterns. Additionally, the chemical composition and antioxidative potential of these EOs were evaluated.

## 2. Results and Discussion

### 2.1. Bacteriological Testing of Milk Samples

A total of 59 milk samples were bacteriologically tested, and pathogens were isolated from 48 (81.35%) samples. The isolated pathogens were the most common mastitis pathogens, including *Streptococcus* spp., *E. coli*, *Cronobacter sakazakii*, *Klebsiella oxytoca*, *Staphylococcus aureus*, *Staphylococcus* spp. coagulase negative, *Streptococcus dysgalactiae*, *Streptococcus* spp., and *Streptococcus uberis*.

The most common among the mastitis associated pathogens were *Streptococcus* spp., which were found in sixteen samples (33.33%), followed by ten samples with *E. coli* (20.83%) and seven samples from which *Streptococcus* spp. β *haemoliticus* was isolated (14.58%). *Staphylococcus aureus* was identified in six samples (12.5%). Furthermore, *Staphylococcus* spp. coagulase negative and *Streptococcus uberis* were found in three samples, each (6.25%), while *Streptococcus dysgalactiae*, *Klebsiella oxytoca*, and *Cronobacter sakazakii* were isolated in one sample each (2.08%).

### 2.2. Antibiotic Susceptibility Testing of Mastitis-Associated Bacteria

There were 21 analyzed antibiotic susceptibility patterns for the most common mastitis pathogens presented in Table 1. Antibiotics included in the antibiotic susceptibility testing were amoxycillin, ampicillin, ceftriaxone, enrofloxacin, erythromycin, lincomycin, neomycin, penicillin, streptomycin, tetracycline, amoxicillin/clavulanic acid, novobiocin, trimethoprim/sulfamethoxazole, and cloxacillin. The current data suggests that the most commonly used antibiotics in mastitis therapy in Serbia are penicillin, streptomycin, gentamicin, tetracycline, cephalexin, sulfonamides, and enrofloxacin [21,22,23]. According to the data obtained in this study, all mastitis associated pathogens are resistant to penicillin, which is not surprising, considering the previously stated fact. All isolates were also resistant to cloxacillin.

Apart from *Streptococcus* spp., all isolated pathogens were sensitive to streptomycin.

### 2.3. EOs’ Chemical Composition Analysis

The detailed chemical composition of the tested oregano and winter savory EOs are listed in the Table 2. A total of 25 of compounds (representing 99.42% of EO) were identified in the oregano EO and 47 (representing 99.66% of EO) in winter savory EO. Aromatic oxygenated monoterpenes (84.56% in *O. vulgare* and 55.34% in *S. montana*) were the most abundant class of compounds in both of the studied EOs. The oregano EO contained high amounts of carvacrol (80.35%) and *p*-cymene (4.82%), followed by thymol (4.21%). Similarly, in the winter savory EO, carvacrol was also the dominant compound (55.01%) while *p*-cymene and γ-terpinene are detected in notable amounts (14.71% and 11.09%, respectively), followed by α-thujene (1.28%), *trans*-β-caryophyllene (2.26%), as well as β-bisabolene (1.51%).

According to the plant list database [24] *O. vulgare* is comprised of the five accepted subspecies (i.e., subsp. *gracile*, subsp. *hirtum*, subsp. *virens*, subsp. *viridulum*, and subsp. *glandulosum*). It has been confirmed that subsp. *gracile* and *hirtum* are carvacrol-rich sources [25]. In addition, the taxon *S. montana* is an extremely polymorphic species with numerous infraspecific systematic categories [26]. Regarding the polymorphism, the EO composition is very variable. According to different authors, several chemotypes are described, but the main types are caryophyllene/geraniol [27], carvacrol [28], and *p*-cymene/geraniol/β-elemene [29] EOs. Furthermore, carvacrol and thymol, or linalool and carvacrol/*p*-cymene chemotypes have been described [15].

The results obtained in this study regarding oregano EOs’ chemical composition agree with the previously published data [25,30], since carvacrol was a dominant compound. Contrary to this finding, the winter savory EO, regarding its main components, does not fit in any of described chemotypes [15,27,28,29] and could possibly represent a chemotype rarely described so far (carvacrol/*p*-cymene/γ-terpinene chemotype).

### 2.4. EOs’ Antioxidant Potential Evaluation

The free radical scavenging capacity (RSC) of the tested EOs of oregano and winter savory, as well as positive control substances evaluated in a series of in vitro tests, are presented in Table 3. All results, except those obtained in the ferric reduction antioxidant potential (FRAP) test, are presented as the IC_50_ values, representing the concentrations of the EOs and positive controls required for neutralization of 50% of free radicals generated. The FRAP test is a different model of antioxidant potential evaluation tests, which correlates with the neutralization of hypochlorite and peroxynitrite anions [31]. Therefore, the results are presented as ascorbic acid equivalents (AAE).

It is well known that oxidative stress represents an inevitable component of many pathophysiological processes. The excessive production of free radicals leads to more intense inflammation process, consequently inducing greater damage of mammary glands in dairy cows [32]. Therefore, agents capable of acting as strong antimicrobials in addition to reducing levels of free radicals would be of great benefit.

The ability of the tested EOs, as well as propyl gallate (PG) and *tert*-butylated hydroxytoluene (BHT), to act as donors of hydrogen atoms or electrons in transformation of DPPH^•^ into its reduced form DPPH-H was tested in the DPPH assay. Although PG (IC_50_ = 0.75 µg/mL) and BHT (IC_50_ = 4.23 µg/mL) exhibited very potent RSC, both EOs were able to reduce the DPPH^•^ into DPPH-H (IC_50_ = 15 µg/mL for *O. vulgare* and 21 µg/mL for *S. montana*). Neutralization of hydroxyl (OH) radicals by oregano and winter savory EOs, as well as the positive control’s antioxidant potential were evaluated by measuring the degradation of 2-deoxyribose caused by OH radicals, generated in a Fenton reaction. Comparing the IC_50_ values obtained for PG (IC_50_ = 8.67 µg/mL) and BHT (IC_50_ = 0.04 µg/mL) with those obtained for tested EOs, it was obvious that the both EOs exhibited lower protective effects on 2-deoxy-D-ribose degradation. The IC_50_ exhibited by EO of oregano was 230 µg/mL, while this value was not determined for winter savory EO in the tested concentration range. None of the evaluated EO managed to neutralize 50% of generated NO radicals. Evaluation of the ability of EOs and the positive control to inhibit the LP pointed to conditionally similar protective effects of BHT (IC_50_ = 7.59 µg/mL) and EO of oregano (IC_50_ = 17 µg/mL). However, winter savory EO exhibited a notably weaker protective effect (IC_50_ = 59 µg/mL). In the FRAP-test, notable antioxidant activity of both tested EOs was detected (35.09 mg AAE/mL for *O. vulgare* EO and 34.41 mg AAE/mL for *S. montana* EO).

Although both tested EOs in most of the assayed systems exhibited modest free radical scavenging effects, it is important to highlight that comparison of antioxidant potential in the present study was performed between pure compounds with confirmed strong antioxidant capacity and EOs. In addition, EOs represent a mixture of different secondary metabolites, some of which do not possess potential to scavenge reactive oxygen species (ROS) and prevent the degradation of biological membranes. Furthermore, BHT, as a synthetic antioxidant, is abused in some pharmaceutical, food, and cosmetic products despite its toxicity [33]. Thus, the usage of EOs in the food and cosmetic industries as a replacement for synthetic antioxidants is suggested by modern trends.

Generally, it has been proven that plants possess significant antioxidant potential, mainly due to the presence of different aromatic, phenolic, and especially flavonoid compounds in the aglycone form. Furthermore, there is an obvious similarity in the recorded RSC between the obtained results for the antioxidant potential of the tested oregano EO and other published data [30,34,35]. Although a generalized comparison of the results published by different laboratories is very difficult considering the different experimental conditions, presentation of the results, different methods of antioxidant potential evaluation, etc., the similar RSC could be explained by the high content of carvacrol in the tested EOs. This aromatic oxygenated monoterpene is, along with thymol, confirmed to exhibit the ability to achieve a resonantly stable radical structure after the donation of a hydrogen atom or electrons to ROS and thus to neutralize the cascade of free radical reactions [36]. Furthermore, due to its non-noxious nature and wide use as a natural preservative in different pharmaceutical, food, and cosmetic products, the Food and Drug administration of the United States (FDA) has considered carvacrol as a safe antioxidant for food stocks [37]. On the contrary, comparison of the antioxidant potential of *S. montana* EO evaluated in this study with other published data is quite difficult since different chemotypes [12,16] are known to exhibit variable biological potential, which is related to the dominant compounds present in the EO. However, the relatively weak antioxidant capacity of winter savory EO examined in this research is in correlation with other published data [38,39].

**Figure 1:**
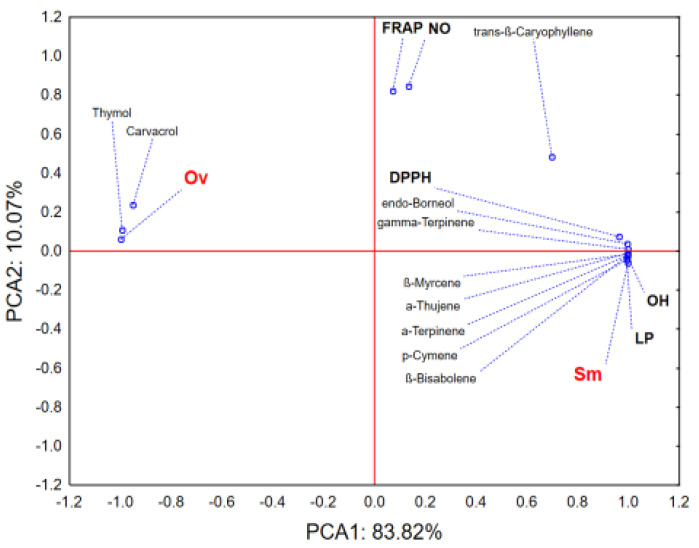
Results of principal components analysis (PCA); Ov—*O. vulgare*; Sm—*S. montana*.

The application of principal components analysis (PCA) on the dataset describing the antioxidant potential and chemical profile of the examined EOs shows that the first two principal components (PCA) describe more than 90% of the samples’ variability (Figure 1). The separate grouping of centroids of the tested EOs samples in the space defined by PCA1 is mostly a result of differences in recorded amounts of thymol and carvacrol, as well as differences in antioxidant potential regarding the scavenging potential of DPPH and OH radicals and inhibition of the lipid peroxidation process. EO of *O. vulgare* (Ov) contains higher amounts of thymol and carvacrol, which also correlates with the stronger antioxidant potential (lower IC_50_ values) recorded in the previously mentioned test systems. On the other hand, *S. montana* EO (Sm) contains higher amounts of γ-Terpinene, *p*-Cymene, α-Thujene, and β-Bisabolene which do not contribute significantly to its antioxidant potential.

### 2.5. EOs Effectiveness against Mastitis-Associated Bacteria

EOs effectiveness against mastitis-associated bacteria is expressed as minimum inhibitory concentrations (MICs) and minimal bactericidal concentrations (MBCs) in Table 4. Antimicrobial activity against the tested mastitis-associated bacteria was demonstrated by both tested EOs. The MIC of *O. vulgare* EO for the tested bacterial species ranged from 0.78 to 6.25 mg/mL, with the lowest MIC values found for the *E. coli* strains. In response to the treatment with *O. vulgare* EO there was no established difference between the tested *E. coli* strains and MBCs ranged from 1.56 to 12.5 mg/mL.

MIC determined for *S. montana* EO ranged from 0.39 to 6.25 mg/mL. The lowest MIC values were found for the tested strain of *Streptococcus* spp. β *haemoliticus*, where MBCs ranged from 0.78–12.5 mg/mL for this EO. The MIC values obtained in our study were comparable to those in the literature. Žitek, et al. [40] investigated the effectiveness of oregano EO obtained by supercritical fluid extraction (SFE) and maceration. The MIC values obtained in their work ranged from 0.147 to 0.327 mg/mL for *S. aureus* and 0.728–2.484 mg/mL for *E. coli*. Compared to these results, the MIC values determined in our work were comparable for four strains of *E. coli* (0.78 mg/mL), while MIC determined for *S. aureus* was higher at 3.125 mg/mL. In research conducted by Kosakowska, et al. [41] oregano EOs and extracts of two different species (Greek oregano and common oregano) exhibited MIC values ranging from 4 mg/mL to 64 mg/mL for *E. coli* and 4–32 mg/mL for *S. aureus*. Maccelli, et al. [42] reported the effectiveness of *Satureja montana* EO against Gram-positive *Listeria monocytogenes*, *Staphylococcus aureus*, *Staphylococcus haemolyticus*, and Gram-negative *Escherichia coli*, *Klebsiella pneumoniae*, *Pseudomonas aeruginosa*, and *Serratia marcescens*. Antibacterial activity of the *Satureja montana* EO examined by Vitanza, et al. [43] showed similar activity toward clinical and reference strains of S. *aureus*, with MIC values in the range 0.39–0.78 mg/mL and MBC of 0.78 mg/mL. In same research, MIC and MBC values for uropathogenic *E. coli* strains were in the range 1.56–3.12 mg/mL.

Several studies have previously demonstrated that carvacrol and thymol have bacteriostatic and bactericidal activity [44,45,46]. Antibacterial activity of the *Satureja montana* EO studied by Vitanza, et al. [43] showed similar activity toward clinical and reference strains of *S. aureus*, with MIC values in the range 0.39–0.78 mg/mL and MBC of 0.78 mg/mL. In same research, MIC and MBC values for uropathogenic *E. coli* strains were in the range 1.56–3.12 mg/mL. The MIC and MBC values obtained in a study conducted by Vitanza, et al. [43] ranged from 0.39 to 6.25 mg/mL which is comparable to the results of our study. Generally, the main constituents of the tested EOs’ are represented by the monoterpenes carvacrol and thymol, which are known for their remarkable inhibitory effects against different pathogens [34,45,47]. Carvacrol and thymol represent structural isomers with a hydroxyl group positioned at different places relative to the phenolic ring [48,49,50]. It is considered that this hydroxyl group increases their hydrophilic ability, which could help them dissolve in the microbial membrane. Furthermore, they have the ability to induce structural and functional alterations by damaging the outer and inner membranes, as well as to interact with membrane proteins and intracellular targets [48,49,50,51]. Lambert, et al. [52] showed that the interaction of thymol with the membrane affects its permeability and results in the release of K^+^ ions and ATP [48,52]. However, the studies have shown that monoterpene hydrocarbons *p*-cymene and γ-terpinene do not exhibit remarkable inhibitory effects against bacteria [45,53].

Earlier research demonstrated that *p*-cymene can enhance the inhibitory effects of carvacrol when these two compounds are used together [34,44,45,47,49,50,52,54]. The authors stated that *p*-cymene is hydrophobic and causes swelling of the cytoplasmic membrane to a greater extent, and that this effect enabled carvacrol to be more easily transported into the cell [55]. Although direct antimicrobial effects of EOs and their main compounds are well documented, their biological effects on cellular function remain obscure. Several studies have shown significant cytotoxic activity of carvacrol and thymol against different cell lines [56,57]. Furthermore, some authors have shown that minor EO components play a modulating role regarding biological potential by producing synergistic effects.

**Figure 2:**
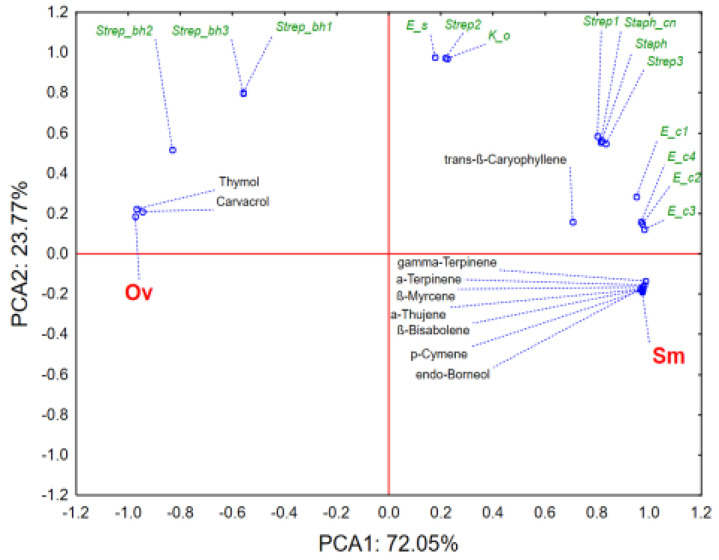
Results of principal components analysis (PCA); OV-*O. vulgare*; SM-*S. montana*.

The application of PCA on the dataset describing antimicrobial potential of the evaluated EOs in relation to their chemical profiles shows that the first two principal components describe more than 90% of the samples’ variability (Figure 2). The separative grouping of samples centroids in the space defined by the PCA1 is mostly a result of thymol and carvacrol quantities, as well as the recorded activity against *Streptococcus* spp. ß *haemolyticus* (Strep_bh), *E. coli* (Ec), *Streptococcus* spp. (Strep), *Staphylococcus* spp. (Staph), and *Staphylococcus* spp. coagulase negative (Staph_cn). It can be noticed that *O. vulgare* EO shows better antimicrobial potential against *E. coli*, *Streptococcus* spp., *Staphylococcus* spp., and *Staphylococcus* spp. *coagulase negative*, possibly as a result of higher thymol and carvacrol abundance. On the other hand, *S. montana* EO is more active against *Streptococcus* spp. ß haemolyticus, which correlates with higher recorded amounts of γ-Terpinene, *p*-Cymene, α-Thujene and β-Bisabolene. Previously published data show that oregano EO can inhibit methicillin-resistant *Staphylococcus aureus* (MRSA) [58]. First, oregano EO affects the permeability of the cell membrane and causes irreversible damage to the cell membrane. Second, the study found that oregano EO can inhibit the respiratory metabolism of MRSA by affecting the metabolites and key enzymes of the TCA cycle. The main component of oregano EO, carvacrol, forms a chimera with DNA. Finally, oregano EO inhibits the expression of the important pathogenic factor pvl in MRSA, thereby reducing the production of PVL toxin. Additionally, oregano EO alone, and in the combination with fluoroquinolones, doxycycline, lincomycin, and maquindox florfenicol could be used to treat infections caused by extended-spectrum β-lactamase (ESBL)-producing *Escherichia coli*. Thus, this may lower, to a great extent, the effective dose of these antibiotics and thus minimize their side effects [59].

## 3. Materials and Methods

### 3.1. Essential Oils

EOs of oregano (*Origanum vulgare* L., Lamiaceae) and winter savory (*Satureja montana* L., Lamiaceae) evaluated in the study were purchased from a certified manufacturer (Pharmanais d.o.o., Babušnica, Serbia). Raw plant material (*Origani herba* and *Saturejae herba*) was sampled before distillation from the manufacturer and after confirmation of identity, voucher specimens (OV-01/2021 and SM-01/2021, respectively) were deposited at the Herbarium of the Pharmacognosy and phytotherapy laboratory, Department of Pharmacy, Faculty of Medicine, University of Novi Sad. According to the certificate obtained from the manufacturer, both EOs were isolated using the internal steam distillation technique (Cellkraft AB, Stocholm, Sweden).

### 3.2. EOs Chemical Composition Analysis

The qualitative and quantitative analysis of EOs was carried out on an HP-5MS capillary column (30 m × 0.25 mm; film thickness 0.25 μm) on an Agilent 6890B gas chromatographer coupled to a flame ionization detector (GC-FID) and mass spectrometry detector (MSD) (5977 MSD, Agilent Technologies Inc., Santa Clara, CA, USA). The samples (1 µL) were injected in split mode (50:1), at inlet temperature of 220 °C. The oven temperature was set at 60 °C and increased at a rate of 3 °C/min up to 246 °C. Helium was the carrier gas (1 mL/min) while the temperature of the MSD transfer line was set to 230 °C. Mass spectral data were collected in scan mode (*m*/*z* = 50–550), while the identification of compounds was performed using NIST (v14) mass spectral database and comparison of relative retention indices (RT), as well as literature data [60].

### 3.3. EOs’ Antioxidant Potential Evaluation

Regarding the complex composition of different plant extracts and EOs, single models are not recommended for the evaluation of their antioxidant potential [61]. Thus, the antioxidant potential of EOs of *O. vulgare* and *S. montana* was evaluated in several in vitro assays. The potential of the EOs to neutralize 2,2-diphenyl-1-picrylhydrazyl (DPPH), hydroxyl (OH) and nitroso (NO) radicals was assessed by previously described spectrophotometric methods [10]. Lipid peroxidation (LP) inhibition potential was evaluated with liposome emulsion used as a test model of biological membranes containing lipids with the Fe^2+^/H_2_O_2_ system of induction. Also, the potential of the essential oils to reduce Fe^3+^ (ferric reduction antioxidant potential, FRAP test) was assessed by the previously described method [10]. As a positive control for antioxidant potential of tested EOs, ascorbic acid (AA), propyl gallate (PG) and *tert*-butylated hydroxytoluene (BHT) were evaluated under the same experimental conditions.

### 3.4. Sampling Procedure

The milk samples were collected at four dairy farms located in Serbia, with 20 to 300 Holstein-Friesian cows per farm. The samples were taken from lactating cows with clinical or subclinical mastitis, without other health problems. Clinical mastitis was assessed by clinical examination, while subclinical mastitis was confirmed using somatic cell count in the milk samples.

Milk for bacteriological testing was sampled aseptically from all animals (with clinical and subclinical mastitis), during the morning milking. The samples were collected in marked sterile tubes and stored and transported at 4 °C to the Laboratory for Milk Hygiene at the Department of Veterinary Medicine, Faculty of Agriculture, University of Novi Sad. The samples were incubated for 48 h at 37 °C on 2% blood agar, using a platinum loop (0.01 mL). For further determination of microorganism’s growth, biochemical and cultural characteristics were taken into account. Isolation and identification of bacterial strain from milk samples were conducted using microbiological procedures to diagnose mammary gland infection published by the National Mastitis Council [62]. A loopful milk sample was streaked on blood agar (Oxoid) and then subcultured on the following selective media: mannitol salt agar, Edwards agar, Salmonella-Shigella agar, and MacConkey agar. The plates were incubated aerobically at 37 °C for 24 h and then examined for colony morphology, pigmentation, and hemolytic characteristics at 24–48 h. For distinguishing staphylococci and other Gram-positive cocci, the catalase test, mannitol fermentation test, coagulase test (either positive or negative), hemolytic pattern, and colony morphology were used. The isolates were confirmed by biochemical tests: oxidase activity, acid production (lactose sucrose and glucose fermentation), indole production, Voges–Proskauer, and hydrogen sulfide production. In addition, each strain was confirmed using Analytical Profile Index API-20 tests (API, bioMeraux, Craponne, France). Staphylococci were isolated on the following media: blood agar, nutrient agar, Ziehl–Neelsen, MSA; for *E. coli* isolation nutrient agar, MacConkey agar, and API 25 were used. Regarding the phenotypic characteristics, the occurrence of α and β hemolysis was used for staphylococci, while pink colonies with precipitation were used for *E. coli.* Edwards agar and hydrolysis of esculin were used for streptococci determination.

### 3.5. Antibiotic Susceptibility Testing of Mastitis-Associated Bacteria

The antibiotic susceptibility patterns for the 16 mastitis-associated bacteria were established in vitro, following the Kirby–Bauer disc diffusion method, on Mueller–Hinton agar (Oxoid) [63]. Antibiotic susceptibility testing was conducted using commercially available antibiotic disks (Bioanalyse) in the following concentrations: ampicillin (10 µg); streptomycin (10 µg); gentamicin (10 µg); trimethoprim/sulphamethoxazole (1.25/23.75 µg); enrofloxacin (5 µg); and ceftriaxone (30 µg). The isolates and reference strains were inoculated on nutrient broth separately and incubated aerobically at 37 °C. After overnight incubation, the bacterial suspension was vortexed and diluted to a turbidity equivalent to that of 0.5 McFarland standards. The bacterial suspension was then spread onto the surface of the Mueller–Hinton agar to make confluent growth. Antibiotic discs were immediately placed on the surface of the agar plate using forceps and incubated aerobically at 37 °C for 16 h. Inhibition zones for various isolates were measured and interpreted as sensitive, intermediate, or resistant according to the Clinical Laboratory Standards Institute (CLSI) [64].

### 3.6. EOs’ Effectiveness Determination against Mastitis-Associated Bacteria

The EOs’ minimum inhibitory concentration (MIC) and minimal bactericidal concentration (MBC) were determined according to a modified resazurin microtiter-plate assay [17]. Briefly, EOs were dissolved in Muller–Hinton broth (MHB) supplemented with 0.5% Tween 80 (Polyoxyethylenesorbitan monooleate, HiMedia Laboratories Pvt. Ltd., Mumbai, India), and diluted to the concentration ranging from 1000 to 0.9 mg/mL. Twenty microliter aliquots of each tested EO were added to 96-well microtiter plates. Subsequently, aliquots of 160 µL of MHB were added to each well. As the final step, 20 µL of the standardized bacterial suspension was inoculated into each well. The test was performed in a total volume of 200 µL with final EOs’ concentrations ranging from 100 to 0.09 mg/mL, while the final microbial concentration was 107 × 10^7^ CFU/mL). The plates were incubated at 37 °C for 24 h. Simultaneously, the same tests were performed for growth control (MHB + test organism), negative control (MHB + solvent + test organism), and sterility control (MHB + test oil). At the end of incubation time, 10 µL of the resazurin solution (0.01%) (Sigma-Aldrich, St Louis, MO, USA) was added to each well. Afterward, the plates were further incubated at 37 °C for 6 h (in darkness). After visual examination, the plates were additionally incubated for 18 h. The change of color from blue (oxidized) to pink (reduced) indicated the growth of bacteria. Finally, wells without the color change (blue color of resazurin remained unchanged) were scored as above the MIC value. MIC was defined as the lowest concentration at which the color had not yet changed. The wells showing complete absence of growth were identified and 100 µL of the solutions from each well was transferred to plate count agar plates (PCA) (Lab M, International Diagnostics Group Plc, Bury, Lancashire, UK) and incubated at 37 °C for 24 h. MBC was defined as the lowest concentration of the EOs at which 99.9% of the inoculated bacteria were killed.

### 3.7. Data Analysis

All the obtained data were processed by Microsoft Office Excel (v2019) and Statsoft Statistica (v12.5) (Tulsa, Oklahoma). Data were analyzed by means of univariate and multivariate statistical methods. The principal components analysis was performed on a dataset describing antimicrobial potential (MIC and MBC), antioxidant potential evaluated in different test-systems (IC_50_ values, except in case of FRAP test) and chemical profile of the examined essential oils (compounds with abundance higher than 1%).

## 4. Conclusions

The current study revealed that, considering in vitro antimicrobial activity against mastitis associated pathogens, oregano and mountain savory EOs could represent a possible solution in mastitis treatment. Furthermore, implementation of EOs in non-antibiotic mastitis treatment could be used in the discovery of new non-antimicrobial strategies used in mastitis control by development of phytopharmaceuticals for intramammary application, as additional or replacement therapy to conventional antibiotic treatment with the possibility to affect the overall antibiotics use. Nevertheless, the transfer of in vitro knowledge to in vivo models is always challenging and will represent the focus of our further studies, bearing in mind the importance of limiting antibiotic use in cattle herds.

## Figures and Tables

**Table 1:** Antibiotic susceptibility patterns for the mastitis-associated bacteria (S—sensitive, I—intermediate, R—resistant). AMX, amoxycillin; AMP, ampicillin; CRO, ceftriaxone; ENR, enrofloxacin; ERY, erythromycin; LIN, lincomycin; NEO, neomycin; PEN, penicillin; STR, streptomycin; TET, tetracycline; AMC, amoxicillin/clavulanic acid; NB, novobiocin; SXT, trimethoprim/sulfamethoxazole; CLO, cloxacillin. *Streptococcus* spp. β heamoliticus (Strep_bh), *Streptococcus* spp. (Strep), *Staphylococcus* spp. coagulase negative (Staph_cn), *E. coli* (E_c), *Klebsiella oxytoca* (K_o), *Enterobacter sakazakii* (E_s), *Staphylococcus aureus* (Staph_a), *Streptococcus dysgalactiae* (Strep_d), and *Streptococcus uberis* (Strep_u).

Bacterial Strains Culture	AMX	AMP	CRO	ENR	ERY	GEN	LIN	NEO	PEN	STR	TET	AMC	NB	SXT	CLO
Strep_bh	S	S	S	S	S	R	S	R	R	S	S	S	S	R	R
Strep	R	R	R	R	R	I	R	R	R	S	R	R	R	R	R
Strep	R	R	R	R	R	I	R	R	R	S	R	R	R	R	R
Staph_cn	S	S	I	S	S	S	S	S	R	S	S	S	S	S	R
Strep	R	R	R	R	I	S	R	R	R	S	R	R	R	R	R
Strep_bh	I	R	S	S	R	S	R	S	R	S	I	S	I	S	R
E_c	R	R	R	S	R	S	R	S	R	S	R	R	R	S	R
E_c	R	R	R	S	R	S	R	S	R	S	I	R	R	S	R
Strep_bh	R	R	S	S	R	S	R	S	R	S	R	S	R	R	R
K_o	R	R	S	S	R	S	R	S	R	S	R	R	R	S	R
E_c	R	R	R	S	R	S	R	S	R	S	I	R	R	S	R
Strep	R	R	I	S	R	S	I	R	R	S	R	S	R	R	R
E_c	R	R	R	S	R	S	R	S	R	S	R	R	R	S	R
E_s	R	R	R	S	R	S	R	S	R	S	S	R	R	S	R
Staph_a	I	R	S	S	S	S	S	S	R	S	S	S	S	S	R
E_c	I	R	S	S	R	S	R	S	R	S	I	S	R	S	R
Strep_u	S	S	I	S	I	S	R	R	R	S	S	S	R	R	R
E_c	I	R	S	S	R	S	R	S	R	S	S	S	R	S	R
Staph_a	I	R	S	S	S	S	S	S	R	S	S	S	S	S	R
Strep_d	S	R	R	S	I	I	R	R	R	S	R	S	I	R	R
Strep	S	S	S	S	S	S	R	R	R	R	R	S	R	R	R

**Table 2:** Chemical composition of *Origanum vulgare* (Ov) and *Satureja montana* (Sm) EOs (%).

Peack No.	Compound	RI *	*O. vulgare*	*S. montana*
Monoterpene Hydrocarbons			3.53	18.47
1	α-Thujene	930	n.d.	1.28
2	α-Pinene	937	0.26	0.81
3	Camphene	952	0.09	0.38
4	β-Pinene	978	0.53	0.83
5	β-Myrcene	991	0.27	1.06
7	α-Phellandrene	1005	0.04	0.23
8	δ-3-Carene	1011	n.d.	0.06
9	α-Terpinene	1017	0.36	2.01
11	Limonene	1030	0.45	0.52
13	β-Ocimene	1037	n.d.	0.06
14	γ-Terpinene	1060	1.53	11.09
16	Terpinolene	1088	n.d.	0.14
Aromatic Monoterpene Hydrocarbons			4.82	14.71
10	p-Cymene	1025	4.82	14.71
Oxygenated Monoterpenes			2.58	3.81
12	1,8-Cineole	1032	0.51	0.32
15	cis-Sabinene hydrate	1070	n.d.	0.24
17	Linalool	1099	0.97	0.81
18	Camphor	1145	0.04	0.05
19	endo-Borneol	1167	0.41	1.04
20	Terpinen-4-ol	1177	0.52	0.75
21	α-Terpineol	1189	0.13	0.26
22	Carvone	1242	n.d.	0.15
27	Geranyl acetate	1382	n.d.	0.19
Aromatic Oxygenated Monoterpenes			84.56	55.34
23	Thymol	1291	4.21	0.33
24	Carvacrol	1299	80.35	55.01
Sesquiterpene Hydrocarbons			2.96	6.96
25	α-Cubebene	1351	0.03	0.05
26	α-Copaene	1376	n.d.	0.16
28	(-)-β-Bourbonene	1384	n.d.	0.15
29	β-Cubenene	1388	n.d.	0.03
30	Longifolene	1408	n.d.	0.51
31	trans-β-Caryophyllene	1419	2.02	2.26
32	β-Copaene	1432	n.d.	0.08
33	γ-Elemene	1433	n.d.	0.03
34	Aromandendrene	1440	n.d.	0.12
35	cis-β-Famesene	1443	n.d.	0.02
36	Humulene	1454	0.24	0.08
37	trans-β-Famesene	1456	0.21	n.d.
38	allo-Aromandendrene	1461	n.d.	0.18
39	γ-Muurolene	1477	n.d.	0.24
40	Germacrene D	1482	0.03	0.58
41	β-Selinene	1486	n.d.	0.21
42	α-Muurolene	1499	n.d.	0.09
43	β-Bisabolene	1509	n.d.	1.51
44	γ-Cadinene	1513	n.d.	0.2
45	δ-Cadinene	1524	0.43	0.46
Oxygenated Sesquiterpenes			0.93	0.35
46	Caryophyllenyl alcohol	1572	0.00	0.06
47	Caryophyllene oxide	1581	0.93	0.27
48	α-Cadinol	1653	n.d.	0.02
Aliphatic Compunds			0.04	0.02
6	3-Octanol	994	0.04	0.02
TOTAL OF IDENTIFIED COMPOUNDS			99.42	99.66

* Retention indices relative to C9-C24 n-alkanes on the HP 5MS column; n.d.—not detected.

**Table 3:** Antioxidant potential of the tested EOs of *O. vulgare* and *S. montana* and positive control substances (AA—ascorbic acid; PG—propyl gallate; BHT—*tert*-butylated hydroxytoluene). FRAP, ferric reduction antioxidant potential; DPPH, 2,2-diphenyl-1-picrylhydrazyl; OH, hydroxyl; LP, lipid peroxidation.

Samples	Assay				
	DPPH IC_50_	OH IC_50_ (µg/mL)	NO IC_50_	LP IC_50_	FRAP (mg AAE */mL EO)
	$\bar{X}$ ** ± SD ***	$\bar{X}\;\pm \;\mathrm{SD}$	$\bar{X}\;\pm \;\mathrm{SD}$	$\bar{X}\;\pm \;\mathrm{SD}$	$\bar{X}\;\pm \;\mathrm{SD}$
*O. vulgare*	15 ± 0.11	250 ± 4.32	n.d. ****	17 ± 0.83	35.09 ± 1.51
*S. montana*	21 ± 0.19	n.d.	n.d.	59 ± 1.73	34.41 ± 2.18
AA	/	20.25 ± 8.39	/	/	/
PG	0.75 ± 0.03	8.67 ± 0.63	/	/	/
BHT	4.23 ± 0.09	0.04 ± 0.01	/	7.59 ± 0.46	/

* Ascorbic acid equivalents; ** Mean value; *** Standard deviation, **** Not detected.

**Table 4:** Minimum inhibitory concentrations (MICs) and minimal bactericidal concentrations (MBCs) of *O. vulgare* and *S. montana* EOs against mastitis-associated pathogens.

Sample	OV * (MIC) (mg/mL)	OV * (MBC) (mg/mL)	SM ** (MIC) (mg/mL)	SM ** (MBC) (mg/mL)
4 strain *E. coli*	0.78	1.56	3.125	6.25
*Cronobacter sakazakii*	6.25	12.5	6.25	12.5
*2 Streptococcus* spp. β *haemoliticus*	3.125	6.25	1.56	3.125
*Streptococcus* spp. β *haemoliticus*	3.125	6.25	0.39	0.78
*Streptococcus* spp.*01*	3.125	6.25	6.25	>12.5
*Streptococcus* spp.*02*	6.25	12.5	6.25	12.5
*Streptococcus* spp.*03*	3.125	6.25	6.25	12.5
*Staphylococcus* spp.*04*	3.125	6.25	6.25	12.5
*Staphylococcus* spp. coagulase negative	3.125	6.25	6.25	12.5
*Klebsiella oxytoca*	3.125	6.25	3.125	6.25

* OV—*O. Vulgare* EO; ** SM—*S. montana* EO.

## Data Availability

The data used to support the findings of this study are available in the present manuscript.
